# A comparative analysis of swallowing accelerometry and sounds during saliva swallows

**DOI:** 10.1186/1475-925X-14-3

**Published:** 2015-01-12

**Authors:** Joshua M Dudik, Iva Jestrović, Bo Luan, James L Coyle, Ervin Sejdić

**Affiliations:** Department of Electrical and Computer Engineering, Swanson School of Engineering, University of Pittsburgh, 3700 O’Hara Street, 15261 Pittsburgh, PA USA; Department of Communication Science and Disorders, School of Health and Rehabilitation Sciences, University of Pittsburgh, 4028 Forbes Tower, 15260 Pittsburgh, PA USA

**Keywords:** Swallowing accelerometry signals, Swallowing sounds, Saliva swallows, Signal characteristics

## Abstract

**Background:**

Accelerometry (the measurement of vibrations) and auscultation (the measurement of sounds) are both non-invasive techniques that have been explored for their potential to detect abnormalities in swallowing. The differences between these techniques and the information they capture about swallowing have not previously been explored in a direct comparison.

**Methods:**

In this study, we investigated the differences between dual-axis swallowing accelerometry and swallowing sounds by recording data from adult participants and calculating a number of time and frequency domain features. During the experiment, 55 participants (ages 18-65) were asked to complete five saliva swallows in a neutral head position. The resulting data was processed using previously designed techniques including wavelet denoising, spline filtering, and fuzzy means segmentation. The pre-processed signals were then used to calculate 9 time, frequency, and time-frequency domain features for each independent signal. Wilcoxon signed-rank and Wilcoxon rank-sum tests were utilized to compare feature values across transducers and patient demographics, respectively.

**Results:**

In addition to finding a number of features that varied between male and female participants, our statistical analysis determined that the majority of our chosen features were statistically significantly different across the two sensor methods and that the dependence on within-subject factors varied with the transducer type. However, a regression analysis showed that age accounted for an insignificant amount of variation in our signals.

**Conclusions:**

We conclude that swallowing accelerometry and swallowing sounds provide different information about deglutition despite utilizing similar transduction methods. This contradicts past assumptions in the field and necessitates the development of separate analysis and processing techniques for swallowing sounds and vibrations.

## Background

Dysphagia is a term used to describe swallowing impairment [1]. It commonly develops as a component of neurological conditions, particularly trauma or stroke [1, 2]. Dysphagia can lead to serious health complications, including pneumonia, malnutrition, dehydration and even death [2, 3]. The current standard of care for swallowing assessment begins with a clinical bedside observation of signs and symptoms, which may be followed up with instrumental examination. Nasopharyngeal flexible endoscopic evaluations involve visualization of the pharynx and upper airway during oral intake, while videofluoroscopic assessment collects dynamic radiographic images of the oral cavity, pharynx, upper airway, and proximal esophagus throughout the entire swallow event [1, 4]. The goal of these assessments is to determine the nature of swallowing pathophysiology, and determine appropriate methods of treatment. Both of these instrumental examinations require skilled expertise and specialized equipment. Previous studies agree that an accurate, simple, non-invasive method of evaluating swallowing function would be a desirable addition to the available tools for assessment.

Efforts to develop less invasive methods of assessing swallowing performance (i.e. whether there is aspiration while swallowing) have been presented over the past two decades, but have demonstrated limited sensitivity and specificity. A variety of non-invasive methods have been explored, including pulse oximetry, surface electromyography and cervical auscultation [5–7]. Cervical auscultation has traditionally been performed using stethoscopes as sensors to detect the sounds of swallowing [8–10]. A high resolution microphone is used to record the sound from the stethoscope before a human perceptually interprets the meaning of the acoustic signal [8–10]. Recently, several studies have been reported using dual-axis accelerometers and algorithms to automatically detect and analyse throat vibrations during swallowing [11, 12]. Although single axis accelerometry recordings are less complicated to analyse, it has been shown that dual-axis accelerometry provides additional information [13]. It is thought that this relates to the fact that hyolaryngeal movements in both the superior and anterior direction are the primary source of the vibrations detected using cervical accelerometry [13]. The source of the swallowing-related signal is likely to be the same for both accelerometer and microphone recordings, but it is possible that the information in each signal is not identical. This would render one technique more of less valuable in detecting specific physiologic abnormalities than the other. Some studies have assumed that swallowing vibrations and sounds are interchangeable [14–18], but there is little evidence to support or disprove this claim as studies which have directly compared the two transducers only investigated very narrow subsets of signal properties [14, 19, 20]. Accelerometers typically operate on the basis of an externally charged, floating capacitor, where one plate is free to move when the device is subject to motion [21]. This capacitor is sealed away from the surrounding environment as it does not need to be exposed to the atmosphere to function [21]. On the other hand, most modern microphones are electret condensers and use a pre-polarized film that is exposed to the surrounding environment in order to generate a signal [22]. To avoid damage to the film caused by prolonged deformation, these devices are often designed in such a way to limit their performance at extremely low (<20 Hz) frequencies. Furthermore, microphones typically operate on and generate an output with pressure waves, rather than vibration direction as with accelerometers, which may result in differences in signal propagation [22]. In addition, these two different types of sensor may differ in size, temperature response, sensitivity, and polar patterns (e.g., [23, 24]) depending on the manufacturer’s specifications. Many microphones in particular utilize a ‘treble boost’ or other non-uniform amplification that improves a signal’s quality with regards to human hearing, but lowers the quality from a digital signal processing perspective. Because of all of these factors, the nature of similarities and differences in swallowing signals obtained using accelerometry versus microphones remains an open question.

During the clinical evaluation of dysphagia, the speech language pathologist conducting the assessment must begin to evaluate swallowing safety using the least harmful swallowing condition available. Humans naturally produce and swallow as much as 1.5 litres of saliva per day, and saliva swallows constitute the majority of swallows produced over the course of any given day in healthy individuals. As a result, saliva swallows are arguably the easiest and safest swallows to execute and are commonly implemented during swallowing assessments. Furthermore, unlike when swallowing liquid or solid boluses, saliva swallows do not introduce external variables, such as bolus viscosity or volume, to the system. As of this writing, there has not been sufficient research to elucidate the effect of these swallowed bolus variables on variability in cervical auscultation signals, and so their effects on swallowing physiology and functionality cannot be independently accounted for. In summary, saliva swallows are the most common swallowing action with the least risk to the patient and the fewest external variables. Therefore, to maximize the benefit of our research, we chose to investigate cervical auscultation exclusively in the context of saliva swallows.

The contributions of this paper are twofold. First, this paper analyses simultaneously recorded swallowing accelerometry signals and swallowing sounds during simple swallowing tasks performed by healthy subjects. These recordings are utilized to understand the differences between the two signal acquisition modalities in the context of cervical auscultation and are studied in time, frequency and time-frequency domain using advanced signal processing algorithms. Second, we also investigate age and sex effects on the extracted signal features for both signal acquisition modalities. There are some notable differences in the anatomy of the neck and throat between the sexes, particularly in the size of the entire upper aero-digestive tract, which could affect either type of recording [25, 26]. Likewise, swallowing performance is known to change with age and could affect the nature of the recorded signals [27].

## Methodology

### Data collection

**Figure 1:**
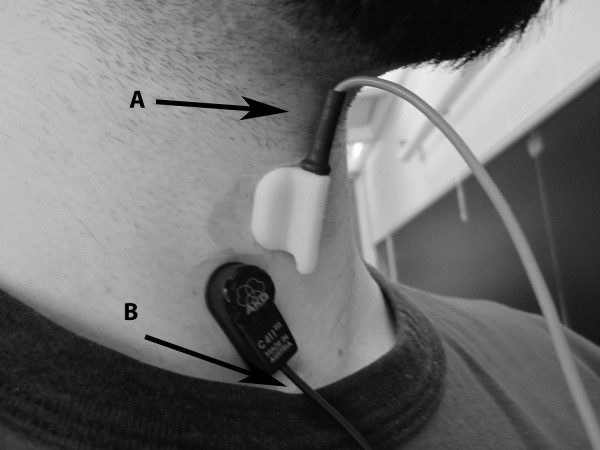
**Transducer mounting locations.** Location of recording devices during data collection. A: Thyroid cartilage B: Top of the suprasternal notch For reference, the microphone (lower device) is approximately 10x30 mm and the accelerometer (upper device) is aligned with the centre axis of the neck.

**Figure 2:**
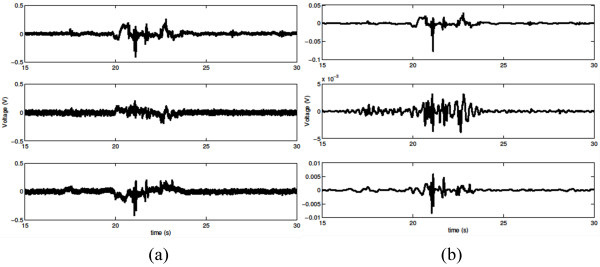
**Pre- and post-processing waveforms.** A single swallow simultaneously recorded with both a microphone and dual axis accelerometer. The top graph is the signal recorded by the microphone while the middle graph is from the anterior-posterior accelerometer axis and the bottom is from the superior-inferior axis. Part **(a)** shows the raw device outputs while part **(b)** shows the same signals after our filtering techniques are applied. One can see obvious differences between sound and vibratory signals as well as different vibration directions.

Our recording equipment consisted of a dual-axis accelerometer and a contact microphone attached to the participant’s neck with double-sided tape. The accelerometer (ADXL 322, Analog Devices, Norwood, Massachusetts) was mounted in a custom plastic case, and affixed over the cricoid cartilage in order to provide the highest signal quality [15]. Its placement can be seen in Figure 1. The two accelerometer axes were aligned parallel to the front of the neck (approximately parallel to the cervical spine) and perpendicular to the same surface (approximately perpendicular to the coronal plane). The sensor was powered by a power supply (model 1504, BK Precision, Yorba Linda, California) with a 3V output, and the resulting signals were bandpass filtered from 0.1 to 3000 Hz with ten times amplification (model P55, Grass Technologies, Warwick, Rhode Island) as swallowing vibrations have been shown to be band-limited to approximately this range [28]. The voltage signals for each axis of the accelerometer were both fed into a National Instruments 6210 DAQ and recorded at 40 kHz by the LabView program Signal Express (National Instruments, Austin, Texas). This set-up has been proven to be effective at detecting swallowing activity in previous studies by maximizing the signal-to-background-noise ratio [11, 28]. The microphone (model C 411L, AKG, Vienna, Austria) was placed below the accelerometer and slightly towards the right lateral side of the trachea so as to avoid contact between the two sensors but record events from approximately the same location. This location has previously been described to be appropriate for collecting swallowing sound signals by maximizing the signal-to-background-noise ratio and can be seen in Figure 1 [14, 19]. The microphone was powered by a power supply (model B29L, AKG, Vienna, Austria) set to ‘line’ impedance with a volume of ‘9’ and the resulting voltage signal was sent to the previously mentioned DAQ. Unlike the swallowing vibrations, this signal was left unfiltered as an upper limit to the bandwidth of swallowing sounds has not yet been found. Instead we recorded the entire dynamic range of our microphone signal (10 Hz to 20 kHz) to ensure that we did not lose any important components of our signal. Again, the signal was sampled by Signal Express at 40 kHz. Figure 2 provides an example of these three analogue signals during a swallow.

The protocol for the study was approved by the Institutional Review Board at the University of Pittsburgh and participants were recruited from the neighbourhoods surrounding the University of Pittsburgh campus. One participant’s data was eliminated from our calculations due to mistakes made during recording resulting in a total of 55 participants with useful data (28 males, 27 females. average age: 38.9±14.9). All participants confirmed that they had no history of swallowing disorders, head or neck trauma or major surgery, chronic smoking, or other conditions which may affect swallowing performance. All testing was performed in the iMED laboratory facilities at the University of Pittsburgh.

With their head in the neutral position, each participant was asked to perform five saliva swallows with a few seconds between each swallow to allow for saliva accumulation. The task was completed at a self-selected pace and was not timed or subject to external guidance or manipulation. This resulted in a total of 275 swallows being recorded for analysis. Each unique task was recorded as a separate text file by the Signal Express software and imported into MATLAB (Mathworks, Natick, Massachusetts) for subsequent data processing.

### Data pre-processing

**Figure 3:**
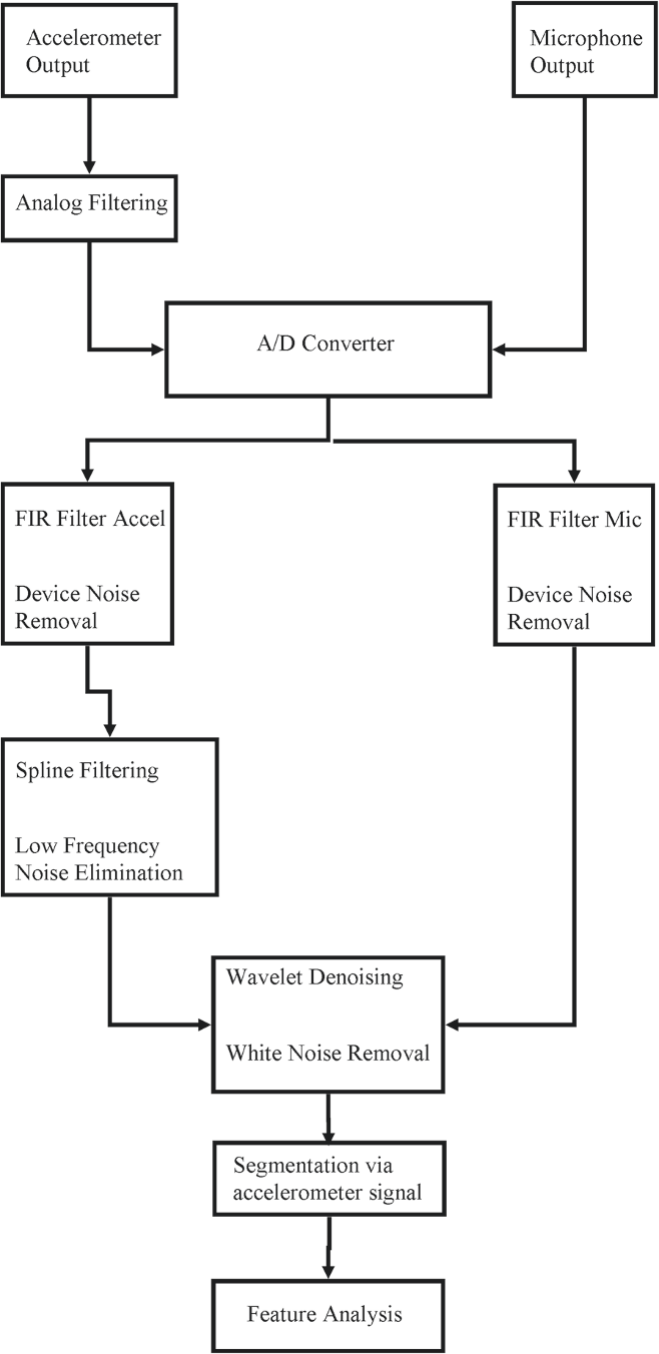
**Pre-processing methodology.** Block diagram of the signal conditioning process used with our data.

Our digital signal processing steps are summarized in Figure 3 and are detailed in the following section.

At an earlier date, the accelerometer’s baseline output was recorded and modified covariance auto-regressive modelling was used to characterize the device noise [29, 30]. The order of the model was determined by minimizing the Bayesian Information Criterion [29]. These autoregressive coefficients were then used to create a finite impulse response filter and remove the recording device noise from our signal [29]. Afterwards, motion artefacts and other low frequency noise were removed from the signal through the use of least-square splines. Specifically, we used fourth-order splines with a number of knots equal to , where *N* is the number of data points in the sample, *fs* is the original 40 kHz sampling frequency of our data, and *f*_*l*_ is equal to either 3.77 or 1.67 Hz for the superior-inferior or anterior-posterior direction, respectively. The values for *f*_*l*_ were calculated and optimized in previous studies [31]. After subtracting this low frequency motion from the signal we denoised the remaining data by using tenth-order Meyer wavelets with soft thresholding [32]. The optimal value of the threshold was determined through previous research to be , where *N* is the number of samples in the data set and *σ*, the estimated standard deviation of the noise, is defined as the median of the down-sampled wavelet coefficients divided by 0.6745 [32]. An example signal after it has been processed by these digital filters is shown in Figure 2. Previous research by Wang and Willett demonstrated a useful method for segmenting data sets into two distinct categories based on local variances [33]. For this study, we applied a modified version of their method and used a proven two-class fuzzy c-means segmentation technique to determine which parts of a given data stream contained vibrations [34]. This allowed us to determine the beginning and end times of each individual swallow for a given subject, and ensured that our feature analysis focused only on the swallowing activity.The device noise filtering algorithm was recalculated with respect to the microphone system and an FIR filter was applied to the swallowing sound signal to eliminate device noise from that signal. We also applied the same 10 level wavelet denoising process to further refine the data. Figure 2 displays an example of this signal before and after it had been filtered. No splines or other low-frequency removal techniques were applied to the swallowing sounds because we had not investigated if such frequencies contained important sound information. We did not develop new segmentation algorithms to extract the five individual swallows from the microphone signal, but instead simply used the time points given by the accelerometer segmentation process.

### Feature extraction

Our next step involved extracting a number of signal features from dual-axis swallowing accelerometry and swallowing sound signals. In the time domain, the signal skewness and kurtosis were calculated using the standard formulas [13, 35]. Finding the swallow duration only required converting the MATLAB indices given in the segmentation step into proper time units.

To calculate information-theoretic features we followed the procedures outlined in previous publications (e.g., [11, 13]). The signals were normalized to zero mean and unit variance, then divided into ten equally spaced levels, ranging from zero to nine, that contained all recorded signal values. We then calculated the entropy rate feature of the signals. This is found by subtracting the minimum value of the normalized entropy rate of the signal from 1 to produce a value that ranges from zero, for a completely random signal, to one, for a completely regular signal [11]. The normalized entropy rate is calculated as
1

where *perc* is the percent of unique entries in the given sequence *L* [11]. In this situation *L* is our recorded signal, a sequence of output voltages from our device. *SE* is the Shannon entropy of the sequence and is calculated as
2

where *ρ*(*j*) is the probability mass function of the given sequence. Lastly the original signal was quantized again, but this time into 100 discrete levels. This allowed us to calculate the Lempel-Ziv complexity as
3

where *k* is the number of unique sequences in the decomposed signal and *n* is the pattern length [36].

Next, in the frequency domain, we determined the bandwidth of the signals along with the centre and peak frequencies. The centre frequency, sometimes referred to as the spectral centroid, was simply calculated by taking the Fourier transform of the signal and finding the weighted average of all the positive frequency components:
4

where *x*(*n*) is the magnitude of a frequency component and *f*(*n*) is the frequency of that component. Similarly, the peak frequency was found to be the Fourier frequency component with the greatest spectral energy. We defined the bandwidth of the signal as the standard deviation of its Fourier transform [11].

We also calculated a number of signal features in the time-frequency domain by utilizing a ten-level discrete Meyer wavelet transform. Previous contributions found that swallowing signals are to some degree non-stationary [37], to which wavelet transforms are better suited than a simple Fourier analysis [13, 38, 39]. Meyer wavelets were used because they are continuous, have a known scaling function [40, 41], and more closely resemble swallowing signals in the time domain compared to Gaussian or other common wavelet shapes [32]. The energy in a given decomposition level was defined as
5

where *x* represents a vector of the approximation coefficients or one of the vectors representing the detail coefficients. ∥∗∥ denotes the Euclidean norm [11]. The total energy of the signal is simply the sum of the energy at each decomposition level. From there, we could calculate the wavelet entropy as:
6

where *Er* is the relative contribution of a given decomposition level to the total energy in the signal and is given as
7

as is detailed in [11].

### Statistical analysis

Our statistical analysis involved transferring the processed features from Matlab to the SPSS (IBM, Armonk, New York) statistical analysis software. There we ran 16 non-parametric Wilcoxon signed-rank tests, eight for each accelerometer axis, comparing the value of each relevant accelerometer attribute against the respective swallowing sound data. The swallow duration was left out of these tests since it was assumed identical for each transduction method. A p-value of 0.003 or less was required for significance after applying the Bonferoni correction. We then ran 25 non-parametric Wilcoxon rank-sum tests, eight for each signal plus one for duration, to investigate possible sex differences in our recordings. In this situation the Bonferoni correction requires a p-value of less than 0.002 for statistical significance. Finally, linear regression curves with respect to age were fitted to the 25 signal features in order to characterize any potential age-related influences on our data.

## Results

**Figure 4:**
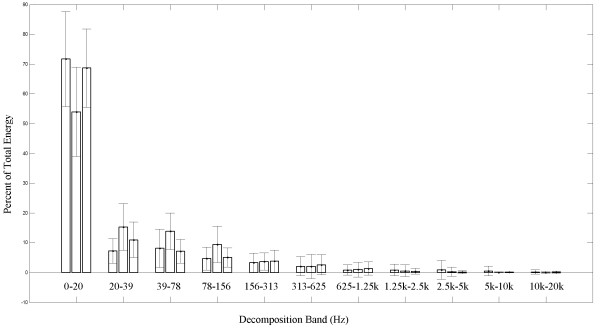
**Mean wavelet decomposition.** Average and standard deviation of the wavelet energy composition of our signals. The left column in each group corresponds to the microphone signal, the middle corresponds to the superior-inferior accelerometer signal, and the right column corresponds to the anterior-posterior signal. The x-axis lists the approximate frequency range of each of the wavelet decomposition levels.

Tables 1, 2, 3 and 4 and Figure 4 summarize the results of our analyses with the mean and standard deviation of each feature.

**Table 1:** **Time domain features in the neutral head position for males**

	A-P	S-I	Sounds
Skewness	-0.369 ± 3.659	0.958 ± 1.703	-0.044 ± 1.037
Kurtosis	49.97 ± 178.1	25.53 ± 55.71	11.97 ± 9.689
Entropy Rate	0.988 ± 0.009	0.989 ± 0.009	0.981 ± 0.030
L-Z Complexity	0.059 ± 0.023	0.071 ± 0.024	0.091 ± 0.091
Duration (s)		2.206 ± 0.948

**Table 2:** **Time domain features in the neutral head position for females**

	A-P	S-I	Sounds
Skewness	-0.194 ± 0.689	-0.123 ± 1.447	-0.095 ± 0.876
Kurtosis	6.607 ± 4.074	20.04 ± 42.31	11.45 ± 21.08
Entropy Rate	0.989 ± 0.004	0.989 ± 0.004	0.988 ± 0.005
L-Z Complexity	0.067 ± 0.018	0.077 ± 0.025	0.081 ± 0.028
Duration (s)		1.833 ± 0.455

**Table 3:** **A summary of frequency domain features in the neutral head position for males**

	A-P	S-I	Sounds
Peak Frequency (Hz)	4.348 ± 14.27	7.730 ± 23.33	24.49 ± 64.95
Center Frequency (Hz)	55.24 ± 116.7	40.80 ± 78.01	150.7 ± 194.9
Bandwidth (Hz)	114.0 ± 231.7	63.69 ± 175.3	420.1 ± 542.5
Wavelet Entropy	1.574 ± 0.606	1.664 ± 0.796	1.463 ± 0.706

**Table 4:** **A summary of frequency domain features in the neutral head position for females**

	A-P	S-I	Sounds
Peak Frequency (Hz)	1.928 ± 1.311	3.976 ± 1.669	14.17 ± 14.21
Center Frequency (Hz)	12.63 ± 11.70	24.14 ± 39.44	110.2 ± 181.8
Bandwidth (Hz)	31.46 ± 34.75	34.32 ± 62.23	320.6 ± 437.1
Wavelet Entropy	1.185 ± 0.586	1.682 ± 0.648	0.997 ± 0.745

We found that, with the exceptions of the anterior-posterior kurtosis (*p*=0.018) and wavelet entropy (*p*=0.006) as well as the superior-inferior L-Z complexity (*p*=0.069) and skewness (*p*=0.255), all accelerometer attributes were statistically different from the corresponding attributes for sounds. The frequency domain features (peak frequency, centre frequency, and bandwidth) were all notably greater for swallowing sounds when compared to the accelerometer signals (*p*<0.001 for all). In the time domain, swallowing sounds had a greater absolute value of skewness than the A-P accelerometer axis while they had less kurtosis than the S-I axis (*p*<0.001 for all). We also noticed that swallowing sounds had a lower entropy rate than both accelerometer signals, but only had a statistically smaller wavelet entropy when compared to the S-I axis (*p*<0.001 for all). Finally, the L-Z complexity of swallowing sounds was determined to be statistically greater than the A-P accelerometer direction only (*p*<0.001).

This study found a number of attributes which differed between male and female participants. In the superior-inferior direction the bandwidth of the accelerometer signal was statistically greater in male participants (*p*<0.001). Meanwhile in the anterior-posterior direction, the centre frequency (*p*<0.001), bandwidth (*p*<0.001), wavelet entropy (*p*=0.001), and kurtosis (*p*<0.001) were all statistically greater in male participants. Swallowing sounds displayed the same sex dependencies as the A-P accelerometer axis signal (*p*<0.001, *p*=0.002, *p*<0.001, and *p*<0.001 respectively).

None of the regression analyses were able to account for more than 5*%* of the variation in any attribute with respect to participant age, with nearly all of them accounting for only 1*%* or less. Data was segregated by the patient’s sex to remove any potential effects from those known differences. Because we used non-parametric statistical tests and obtained a relatively small population sample, we felt that we lacked the necessary power to properly assess the statistical significance of these extremely small effects across our population sample’s age.Figure 4 shows the average energy distribution of the wavelet coefficients of all three signals along with the standard deviation of each level’s energy. Data from all subjects has been included for simplicity and readability. They all show that the vast majority of swallowing wavelet energy is contained in the lowest frequency components. We clearly see that well over 50% of the swallowing sound and vibration energy is contained below 20 Hz with only a minimal amount of energy in the higher frequency bands.

## Discussion

### Age and sex effects on swallowing sounds and swallowing accelerometry signals

All three of our signals demonstrated some amount of sex dependence in the frequency domain. Specifically, the centre frequency of both the microphone and anterior-posterior accelerometer signals was statistically significantly greater in men, while the same was true of the bandwidth of all three signals. The wavelet entropy of the swallowing sounds and anterior-posterior accelerometer signal is also greater in men, resulting in a more chaotic and less predictable signal, and most likely related to the signals’ shifts towards higher frequencies. We suspect that these differences may be due to the sex based variations in the size and position of the laryngeal prominence, since our recording devices were placed just below this structure [25]. This structure tends to protrude further in males, yet undergoes the same motion during a swallow as females [26, 42]. This could produce higher frequency vibrations in male subjects as tissues are displaced faster in the anterior-posterior direction to accommodate the larger moving structure.

In the time domain, we also found that the kurtosis of the A-P accelerometer and microphone signals are greater in men. A higher kurtosis implies that the signal has a greater intensity over a shorter period of time, which conforms to our earlier frequency domain findings as higher frequency pulses tend to have greater kurtosis [35]. This finding adds validity to our previous statistical conclusions.

Our finding that the swallow duration does not vary statistically with regards to sex or demonstrate any notable trends with regard to age runs counter to past research on this subject [43]. Our results are similar to a previous study that used the same automated segmentation algorithm supported by a videofluoroscopic evaluation, suggesting that we can exclude recording errors as the source of the discrepancy [34]. Meanwhile, other studies which reported sex differences on swallowing duration utilized manual inspection of the videofluoroscopic images or vibratory/sound spectrum ([44] and [43] respectively), and reported much shorter durations. We assume, then, that our loss of sex dependence on swallowing duration is a result of processing and segmentation differences between this and prior studies. As the times when a swallow begins and ends are not clearly defined, particularly when concurrent videofluoroscopic imaging is not utilized, this is not surprising. The lack of age dependence in our swallowing duration data is most likely due to our small sample size. Our previous study, which did report statistically significant effects of age, utilized a sample size that was orders of magnitude larger than this study’s sample population. As a result, our past work had more statistical power to detect what our regression analysis in this study suggests was a relatively minor influence [34].

### Comparing swallowing sounds and swallowing accelerometry signals

Our time domain contrasts found only a few statistically significant differences between swallowing vibrations and sounds. We noticed that the anterior-posterior accelerometer signal skewness had a statistically significantly lower value than the acoustic signal. In fact, while swallowing sounds can have either positive or negative skewness, the A-P accelerometer signal had typically negative skewness. This means that swallows produce vibrations in the anterior-posterior direction that slowly increase in intensity before decreasing much faster, whereas swallowing sounds do not follow such a consistent pattern [35]. We also found that the A-P accelerometer signal had a statistically lower Lempel-Ziv complexity when compared to the swallowing sound signals. While the complexity of both signals is already quite low, this indicates that our discretized A-P accelerometer data can be compressed further without losing information about the signal [45].

The last statistically significant time domain comparison we found was the entropy rate, which was lower in both accelerometer signals than in swallowing sounds. However, all three signals displayed entropy close to 1 with only a single mean having a value below 0.99, indicating that all of our discretized signals were highly predictable. While the exact level of regularity varies with each signal, our study shows that both swallowing sounds and accelerometry follow a predictable pattern when the data is discretized to ten levels.

Our frequency feature contrasts are particularly interesting. First, they show that the swallowing sounds contain statistically higher frequency components when compared to either accelerometer direction. This demonstrates the existence of higher frequency features which only one transduction method detected. Second, our results demonstrate peak and centre frequencies that are much lower than those reported in many other studies [10, 15, 28]. Even though we cannot exclude the possibility that our recording technique is the source of the discrepancy, it is more likely due to our use of different experimental set-ups. Several of these past studies utilized recording devices which could not detect sounds below 50 Hz and so would not be able to detect low frequency information to the same extent as our microphone [10, 14, 19]. This is a difference that may be influenced by the manual auditory or spectrogram based segmentation used in other studies [10, 28]. Notably, these studies do not take into account the non-linear nature of human hearing, which generally does not extend below 20 Hz, and may have excluded valuable data from their analyses [46]. Finally, one also cannot ignore the different hardware and transduction methods used in these studies, which would invariably affect both data recording and the resulting analyses [10, 14].

The wavelet energy plots in Figure 4 are distributed as one would expect: a roughly exponentially decaying pattern as frequency increases. Clearly, they show that the overwhelming majority of signal energy is concentrated in the lowest frequency components, particularly for the A-P accelerometry and microphone signals. This is logical, considering the time-scale that swallowing operates on and the temporal dynamics of swallowing [47].

### Future applications and limitations

The results presented in this work will provide tangible benefits to the design of future studies using cervical auscultation. It provides baseline values for a wide selection of features in both the time and frequency domains with regards to healthy swallows. As a result, future investigators will be able to ensure that their chosen hardware is capable of recording their signal effectively. This is especially important when the researcher is choosing whether to utilize a microphone, an accelerometer, or both. While several past studies have assumed that swallowing vibrations and sounds are identical [14–18], this study demonstrates that the transduction modality does significantly affect what data is recorded. Certain filtering or other processing techniques that are useful for one modality may not be applicable to the other, whereas results obtained using sounds might not be comparable to results found with vibration data. Neither device is inherently superior, but instead they each offer different advantages and disadvantages that must be accounted for during the study design process.

In addition, future investigators will also be able to compare their results, presumably recorded from subjects that do have swallowing disorders, to what this study has found with regards to healthy subjects. This could be used to quickly and easily confirm proper function of different data collection systems as well as reduce the need to manually gather data from a control group when investigating pathological effects on the data. If a cervical auscultation based method of screening for dysphagia is to be developed, characterizing normal swallowing function so that it can later be objectively compared to abnormal swallowing is a necessary first step.

The greatest limitation of this study is the population sample size. By using multiple trials we were able to collect a respectable number of individual swallows. However, our sample size of 55 participants may not be a perfect representation of the whole population. The sex and age distributions of our sample were kept relatively even, but other factors such as race were not artificially controlled for and their distribution in our population sample was left to chance. In addition, this study is somewhat limited in its scope. We investigated only saliva swallows made in a neutral head position when many other types of swallows can be made, both in normal life and during a true clinical swallowing examination. While these results are useful due to the common occurrence of saliva swallows, as detailed previously, they may not be an accurate representation of swallows made in a different head position or swallows made with different bolus consistencies.

## Conclusion

In this study, we recorded data from healthy adult subjects performing saliva swallows using both a dual-axis accelerometer and a contact microphone. The nine different time and frequency domain features demonstrated varying degrees of significance with respect to the subject’s sex, while no features demonstrated any notable variation with respect to age. When comparing the swallowing sound features to the accelerometer signals, we found that most of the features were statistically significantly different with the exception of anterior-posterior accelerometer kurtosis and wavelet entropy as well as superior-inferior accelerometer skewness and L-Z complexity. We conclude that despite their similarities, these two methods of transducing swallowing vibrations and sounds provide distinct and complementary information about deglutition.
